# Extracellular matrix and its therapeutic potential for cancer treatment

**DOI:** 10.1038/s41392-021-00544-0

**Published:** 2021-04-23

**Authors:** Jiacheng Huang, Lele Zhang, Dalong Wan, Lin Zhou, Shusen Zheng, Shengzhang Lin, Yiting Qiao

**Affiliations:** 1 Division of Hepatobiliary and Pancreatic Surgery, Department of Surgery, First Affiliated Hospital, School of Medicine, Zhejiang University, Hangzhou, 310003 China; 2 School of Medicine, Zhejiang University, Hangzhou, 310003 China; 3 NHC Key Laboratory of Combined Multi-Organ Transplantation, Hangzhou, 310003 China; 4 Key Laboratory of the Diagnosis and Treatment of Organ Transplantation, Research Unit of Collaborative Diagnosis and Treatment For Hepatobiliary and Pancreatic Cancer, Chinese Academy of Medical Sciences (2019RU019), Hangzhou, 310003 China; 5 Key Laboratory of Organ Transplantation, Zhejiang Province, Hangzhou, 310003 China; 6 Shulan (Hangzhou) Hospital Affiliated to Zhejiang Shuren University Shulan International Medical College, Hangzhou, 310000 China

**Keywords:** Cancer therapy, Cancer therapy

## Abstract

The extracellular matrix (ECM) is one of the major components of tumors that plays multiple crucial roles, including mechanical support, modulation of the microenvironment, and a source of signaling molecules. The quantity and cross-linking status of ECM components are major factors determining tissue stiffness. During tumorigenesis, the interplay between cancer cells and the tumor microenvironment (TME) often results in the stiffness of the ECM, leading to aberrant mechanotransduction and further malignant transformation. Therefore, a comprehensive understanding of ECM dysregulation in the TME would contribute to the discovery of promising therapeutic targets for cancer treatment. Herein, we summarized the knowledge concerning the following: (1) major ECM constituents and their functions in both normal and malignant conditions; (2) the interplay between cancer cells and the ECM in the TME; (3) key receptors for mechanotransduction and their alteration during carcinogenesis; and (4) the current therapeutic strategies targeting aberrant ECM for cancer treatment.

## Introduction

Cancer is a leading cause of death which severely impedes the health career for extension of life expectancy in the world. The incidence and mortality of cancer are increasing year by year. According to the latest global cancer statistics in 2020, 19.3 million new cases were diagnosed and cancer contributed to 10.0 million deaths^1^. Therefore, cancer is becoming one of the most serious problems which threaten public health. The most striking attributes of cancer are uncontrolled proliferation, local invasion, and distant metastasis. Nowadays, the mainstream therapies for cancer treatment include surgery, chemotherapy, radiotherapy, targeted therapy, and immunotherapy. Cancer-related death is mainly caused by tumor recurrence and distant metastasis after systemic antitumor treatment. Although great advances have been achieved for cancer treatment in recent years, especially in the field of targeted therapy and immune therapy, the pursuit for converting this life-threatening disease into a manageable chronic condition has never stopped. The comprehensive understanding of cancer cells, as well as the microenvironment supporting the malignant behavior of cancer cells, are of equal importance for developing novel therapeutics against cancer.

The extracellular matrix (ECM), which comprises the interstitial elements within tissues or organs for all metazoan organisms, plays vital roles for all biological processes by providing architectural support, anchorage for cell adhesion, a reservoir for water, and various growth factors, as well as inductions for intracellular signaling pathways. According to a comprehensive study utilizing both proteomic analyses of the in vivo ECM composition and in silico prediction^2^, 278 genes were identified as core elements of the “matrisome” for humans, accounting for 1% of the entire proteome.

Ever since the identification and characterization of collagen, the most abundant component of ECM, in the 1930s, the complicated network of ECM started to be gradually revealed through modern biochemistry methods^3^. Most of the proteins in the ECM can be classified into two groups, fibrous proteins, and glycosaminoglycan. The former include collagen, fibronectin, elastin, and laminin, and the latter mainly consists of hyaluronic acid, chondroitin sulfate, keratan sulfate, and heparan sulfate. These molecules are crosslinked and distributed heavily in the ECM, forming the mesh structure for tissues. As early as the 1970s, the critical roles of ECM in the determination of cell morphology and responses to growth factors had been proven with solid empirical evidence^4^. Then the pursuit of discovering the intermembrane signal transducers linking ECM and intercellular signaling pathways produced many great works marked by the identification of integrins in 1980s^5–9^. Ever since then, the field of ECM–cell interaction developed rapidly, and the vast signaling network bridging extracellular environment and complicated cell behaviors started to reveal itself gradually due to the continuous efforts of researchers and technological advances. During the 2010s, the clinical application of Ibrutinib (a small molecular compound inhibiting integrin signaling) for the treatment of lymphoid leukemia and lymphoma was a hallmark event for the successful translation of biological knowledge to practical medicines in this research area^10,11^. At the same time, bioengineering of artificial and natural ECM materials also achieved great success in multiple branches of medicine, such as osteology, odontology, dermatology, and ophthalmology. For example, an artificial dermal regeneration template has been invented for the treatment of aplasia cutis congenital, a severer disorder characterized by the congenital absence of skin^12^.

**Fig. 1:**
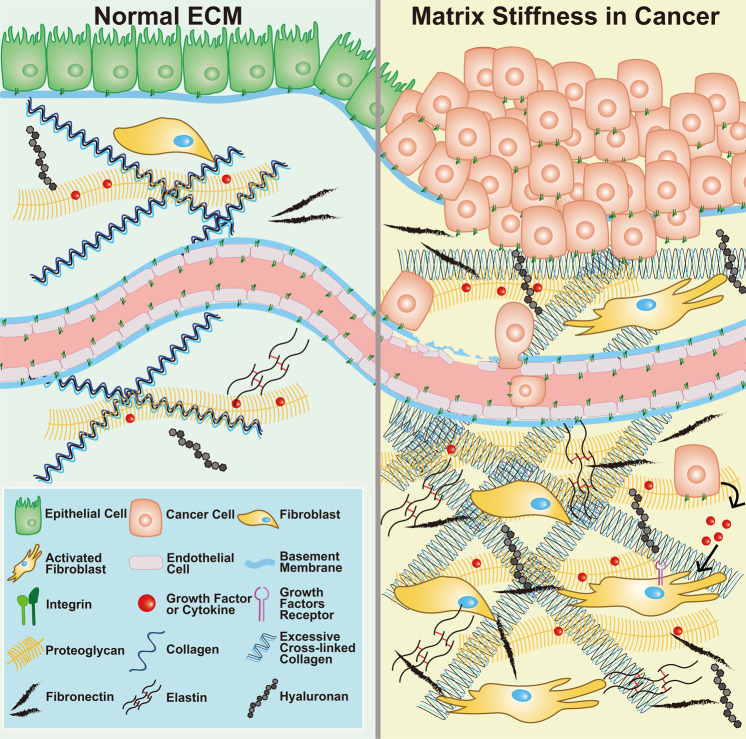
Schematic illustration of ECM components in normal tissue (left) and the TME (right). Matrix stiffness is mainly related to excessive collagen and HA within TME. Both cancer cells and fibroblasts contribute to the remodeling of the ECM during its stiffness, fundamentally influencing many critical biological processes during the development of cancer

As one of the major components of the tumor microenvironment (TME), the dysregulation of ECM is a remarkable feature of cancer (Fig. 1). During the development of cancer, malignant cells contribute to ECM stiffness, and, in return, the stiffened ECM alters the characteristics of cancer cells. The communication between cancer cells and the ECM activates several vital pathways related to mechanotransduction. Therefore, a comprehensive understanding of the dysregulation of the ECM in the TME would contribute to the discovery of promising therapeutic targets for cancer treatment. In the present review, the structures and functions of multiple ECM components, such as collagen, fibronectin, elastin, and so on, were introduced. Then we summarized their alterations and the underlying mechanisms during matrix stiffness in cancer. Meanwhile, the downstream biological effects of matrix stiffness on both cancer cells and other cells in TME were also discussed. Subsequently, several pivotal receptors for ECM and their roles in malignant transformation were summarized. Afterward, both clinical and preclinical therapeutic applications of ECM-related signaling for cancer treatment were discussed in-depth based on our current knowledge from basic researches and clinical studies. Finally, the vision and several potential Gordian Knots for targeting ECM-related signaling for cancer treatment were summarized and discussed to call for more attention to this research field.

## Major ECM components: structure and function

### Collagen

Collagen makes up most of the ECM, accounting for approximately 90% of the ECM and 30% of the total protein in humans^13^. Currently, 28 types of collagens have been identified, encoded by 43 genes^14^. All collagens are homotrimers or heterotrimers of three polypeptide chains (α chains), comprising numerous Gly-X-Y repeats, X and Y being frequently proline and 4-hydroxyproline, respectively^15^. Glycine provides conformational flexibility, while proline provides conformational rigidity. Therefore, the rod-shaped triple helix is stabilized by interchain hydrogen bonds and electrostatic interactions^13^. α Chains vary greatly in size (ranging from 662 up to 3152 amino acids for the human α1 (collagen type X) and α3 (collagen type VI) chains, respectively) as well as the frequencies of imperfections and interruptions of Gly-X-Y repeats, resulting in differences in plasticity, flexibility and recognition patterns among various types of collagens^13^.

A more intensive collagen mesh is constructed based on the posttranscriptional product for collagen maturation. Collagen forms macromolecules by intermolecular cross-linking^16^. The cross-linked collagen provides tissue intensity and tenacity. For example, the mechanical properties of fibril-forming collagen are largely dependent on the extent of covalent cross-links within and between triple helixes, including disulfide bonds, the Nε(γ-glutamyl)lysine isopeptide, reducible and mature cross-links produced via the lysyl oxidase pathway, advanced glycation end products, hydroxylysine-methionine cross-links, and arginyl ketoimine adducts called arginoline^17^. Moreover, the existence of noncollagenous domains in collagens that can assemble mutually between collagens or even between collagens and other ECM proteins increases the complexity of such supramolecules^18^. Thus, collagens can form fibrils, beaded filaments, anchoring fibrils, and even networks^19^.

Collagens are long-lived proteins due to their high glycation level, but their degradation is still critical for both normal turnover and pathological destruction of connective tissue^20^. Matrix metalloproteinases (MMPs) participate in the physiological and pathological degradation of collagens. These zinc-dependent endopeptidases comprise a large family of 28 members^21,22^. For example, MMP-1, MMP-8, MMP-13, and MMP-14 cleave fibril-forming collagens I, II, and III, while MMP-2 and MMP-9 cleave denatured collagens and collagen IV^14^. Sheddases are another family of enzymes that digest collagens^14,23^. During the digestion of MMPs, signaling molecules such as endostatin and tumstatin are released from collagens, simultaneously leading to the alteration of mechanical characteristics as well as signaling transduction in the microenvironment^24,25^. As the major component of the ECM, the amount and posttranslational modifications of collagens often undergo tremendous alterations during the development of cancer, resulting in a fundamental influence on the behavior of cancer cells and other cells in the TME, features that would be discussed later in this review.

### Fibronectin

Fibronectin is low in abundance but has diverse functions in the ECM. Soluble fibronectin is secreted by hepatocytes and into the circulation^26,27^, while the insoluble form is produced by a variety of mesenchymal cells, including fibroblast^28^ and endothelial cells. In the vasculature, vascular smooth muscle cells are a major producer of fibronectin^29^.

Fibronectin is a type of dimer proteoglycan, which is interwoven with two subunits via a disulfide bond at the C-terminus^30,31^. A fibronectin subunit weighs approximately between 220 and 250 kD^32^. There are several structural domains in both subunits, and the structural domains mainly consist of three structural modules, 12 repeat fibronectin type I, 2 repeat fibronectin type II, and 15–17 repeat fibronectin type III^33–35^. These structural domains constitute the functional domains of fibronectin, including a domain that weighs 70 kD at the N-terminus (fibronectin type I_1–9_), a central binding domain (CBD) that weighs 120 kD (fibronectin type III_1–12_), and a heparin-binding domain (HepII) (fibronectin type III_12–14_).

Fibronectin has profound effects on cell adhesion, migration^36^, proliferation^37,38^, blood coagulation^39,40^, vascularization^41–43^, clearance of bacteria by phagocytes^44^, and wound healing^45,46^, among others. In the ECM, fibronectin connects various structural proteins to form an integrated matrix, such as collagens^47^, fibrillin^48^, and tenascin-C^49,50^. For example, the antibody targeting the collagen-binding site in fibronectin could suppress the fibrillogenesis of collagen, suggesting that type I collagen cannot assemble without fibronectin^51^.

Other than binding to multiple structural proteins to reinforce the ECM, fibronectin directly interacts with many other proteins to exert regulatory functions^52–54^. First, fibronectin contains abundant arginine–glycine–asparagine (RGD) sequences that can recognize and bind to integrins on the cell membrane^55^. Therefore, fibronectin has a profound effect on intracellular signaling transduction by inducing integrin attachment. For example, the interaction of MMP-9-degraded fibronectin and integrin αvβ6 leads to aggressive migration and invasion via ERK1/2 and PI3K/AKT/Smad-1/5/8 pathways in breast cancer^56^. By contrast, many growth factors can directly interact with fibronectin. For example, insulin-like growth factor (IGF), fibroblast growth factor (FGF), transforming growth factor-beta (TGF-β), hepatocyte growth factor (HGF), and platelet-derived growth factor (PDGF) can interact with the fibronectin domain^57–60^. FGF, vascular endothelial growth factor (VEGF), and PDGF can bind to the heparin II domain in fibronectin^58^, and PDGF can attach to the fibronectin first type III repeat (FNIII1)^57^. Moreover, extra domain A (EDA) in fibronectin can increase VEGF-C expression in colorectal carcinoma^61^. Therefore, although fibronectin is low in abundance in the ECM, it plays a vital role during malignant transformation.

### Elastin and laminin

Elastin is the primary component of elastic fibers and is mainly found in ligaments and vascular walls. Elastin maintains the tenacity and intensity of tissues along with collagen by rebelling against tissue deformation or rupture. Compared with collagen, elastin is highly resilient because of its amino constituents and dynamic three-dimensional (3D) structure. Glycine makes up one-third of the polypeptide, and proline accounts for approximately 10%, while hydroxyproline accounts for less than 1%. The β-turn in the polypeptide chain is produced based on the interaction of Gly_4_ (N–H) and Gly_1_ (C = O) or Leu_5_ (N–H) and Val_2_ (C = O), resulting in the resilience of elastin.

Laminin, together with collagen, makes up the constituents of the basement membrane. Therefore, laminin is involved in vascularization, especially in the process of vessel maturation^62^. During re-epithelialization in wound healing, laminin is upregulated to provide an interface for the adherence of epithelial cells to adhere and stretch^63^. Laminin is polymerized by three different chains, one α chain, one β chain, and one γ chain, which are encoded by separate genes^64,65^. Five forms of α chains (LAMA1-5) and three forms of β chains (LAMB1-3) and γ chains (LAMC1-3) can be found in laminins^66^. For example, laminin comprising α2β2γ1 is named laminin-221. The laminin network is constructed along with collagen type IV, fibronectin, and perlecan in basement membranes^63,67^.

### Hyaluronic acid

Hyaluronic acid (HA), another primary component in ECM, is a high-molecular-weight glycosaminoglycan comprising disaccharide repeats of N-acetylglucosamine and glucuronic acid^68^. HA is synthesized by the alternative addition of glucuronic acid and N-acetylglucosamine to the growing chain, using their activated nucleotide sugars (uridine diphosphate glucose (UDP) and UDP-N-acetylglucosamine) as substrates. This reaction is mediated by hyaluronan synthase 1, 2, and 3 (HAS1–HAS3) localized on the cell membrane. The enzymatic degradation of HA is mediated by hyaluronidase, b-d-glucuronidase, and β-N-acetyl-hexosaminidase^69^. The number of repeated disaccharides in a completed HA molecule can reach 10,000 or even more. Its long polymer chains form random coils entangled in solution, and its numerous hydroxyls capture a huge quantity of water by forming hydrogen bonds^70^. Therefore, HA mechanically increases the elastoviscosity in the ECM.

In addition to its unique viscoelastic nature, HA functions as an important “reservoir” for water, buffering ion exchange, water, and osmotic balance within the ECM. Moreover, some substances and biomacromolecules are selectively permeable to HA due to their charged surface and selective domains. Therefore, HA can serve as a sieve: particles with a huge molecular size are hindered and immobilized, while smaller molecules tend to pass through HA more efficiently^71^. Additionally, HA can be recognized by various types of cells through membrane receptors such as CD44 and receptor for hyaluronan-mediated motility (RHAMM), as well as intracellular signaling transducers such as cell division cycle 37 (CDC37)^72^, P-32^73^, and hyaluronan binding protein 4 (HABP4)^74^. Such recognition plays vital role in many biological procedures, including cell mobility, invasion, proliferation, and inflammation.

### Chondroitin sulfate, keratan sulfate, and heparan sulfate

Chondroitin sulfate, keratan sulfate, and heparan sulfate are heteropolysaccharides that also belong to the class of glycosaminoglycans^75^. Their molecular structures are similar to that of HA and comprise repeated disaccharides. The difference among HA, chondroitin sulfate, keratan sulfate, and heparan sulfate lies in the carbohydrate of the monomer and sulfate ester position. The carbohydrates that comprise chondroitin sulfate are [→4GlcAb1→3GalNAcb1→], and the sulfated site could be position 4 (CS A, chondroitin-4-sulfate) or position 6 (CS C, chondroitin-6-sulfate) of the repeating unit^76,77^. Keratan sulfate comprises [→3Galb1→4GlcNAcb1→], and sulfate esters can be found at C-6 in one carbohydrate or both monosaccharides with a hydroxyl group^78^. Heparan sulfate comprises [→4GlcAb1→4GlcNAca1→]. O-sulfation modification of heparan sulfate mainly occurs at C-2 of iduronic acid (IdoA) and C-6 of glucosamine, and sometimes at C-2 of GlcA and C-3 of glucosamine^79^. Various sulfate group sites lead to the heterogeneity of the glycosaminoglycan structures and functions^75^.

These three types of glycosaminoglycans described above have the following characteristics. First, glycosaminoglycans bind to proteins by covalent bonds^80^. For example, chondroitin sulfate can bind to matrix proteins, growth factors, cytokines, chemokines, and protease inhibitors^81–83^. Second, the sulfate substituent on the carbohydrate can mediate the coupling between glycosaminoglycans and metal ions, thus preventing the formation of peroxide catalyzed by metal ions. Finally, the hydrogen bond is formed when glycosaminoglycans interact with water, leading to the mechanical effect of viscoelasticity.

## Fibroblasts

Stromal cells, including fibroblasts and pericytes, are the major source of ECM^84^. Fibroblasts are widely distributed in most connective tissues such as the bone marrow, lymph nodes, ovaries, and solid tumors^85^. Pericytes are specifically located surrounding the endothelial cells on the interior surface of blood vessels^86,87^.

Fibroblasts are one of the major cell types within the TME in terms of both number and function. Some studies have shown that fibroblasts account for 70 to 90% of the whole tumor volume of breast cancer and pancreatic cancer^88–90^. More importantly, fibroblasts play a central role in the formation and turnover of ECM for two reasons. First, fibroblasts directly produce structural macromolecules, such as collagen, fibronectin, and laminin^91^. Second, enzymes involved in the modification and degradation of these structural macromolecules are also secreted by fibroblasts such as lysyl hydroxylases and metalloproteinases^92,93^.

Fibroblasts are regulated by many signals, including cytokines, chemicals, and environmental signals, such as heat and mechanical forces, thus contributing to ECM remodeling. For example, TGF-β can enhance the production of both collagen and fibronectin, as well as procollagen lysyl hydroxylase 2 (LOX2), while tumor necrosis factor-alpha (TNF-α) can inhibit collagen synthesis in fibroblasts^92,94^. Moreover, TNF-α and interleukin (IL)-1 can induce the production of MMP-1, -3, and -9 by fibroblasts, leading to the degradation of collagen in the TME^94^. Interestingly, primary human dermal fibroblasts proliferate faster and produce more collagen on amine-rich (NH3) surfaces when cultured in vitro compared with surfaces coated with carboxyl acid (COOH) and hydrocarbon (CH3)^95^. Similarly, the collagen levels of human patellar tendon fibroblasts, cardiac fibroblasts, and periodontal ligament fibroblasts are all enhanced by repeated mechanical stretching^96–98^. Moreover, repeated mild heat shocks have been shown to increase dermal fibroblast activity and collagen production^99^. These lines of evidence suggest that fibroblasts are highly flexible cells that convert signals from multiple sources into changes in ECM components.

The ECM is an orchestration of many components, including but not limited to matrix proteins, glycosaminoglycans, growth factors, enzymes, and fibroblasts, whose balance is critical to maintaining tissue homeostasis. For cancer, which is a complicated disease involving active interaction between cells and their microenvironment, ECM stiffness is a distinctive feature and a promising therapeutic target (Fig. 1).

## Matrix stiffness in cancer: phenomena, mechanisms, and biological effects

### Alterations of tissue stiffness in cancer

Stiffness is defined as the extent of deformation when the external force is applied to an object or material^100,101^. For most tissues without bones, their stiffness is largely dependent on the quantity and components of the ECM. Tumors frequently exhibit higher stiffness than normal tissues. For example, an elastography study of breast cancer demonstrated that the stiffness of tumor tissue is higher than that of normal tissue^102^. In another study, in vivo shear-wave elastography analysis of 337 breast cancer patients also revealed that tissue stiffness values are positively correlated with malignant phenotypes, including larger tumor sizes, higher histologic grades, and estrogen receptor (ER) status, with triple-negative breast cancer tissues ranking stiffest^103^. Similarly, a study of 373 patients with focal liver lesions showed that the mean stiffness values of hepatocellular carcinoma, intrahepatic cholangiocarcinoma, and metastasis were 34 (range: 4.4–188), 25 (range: 5.5–79), and 30 (range: 4.7–64), respectively, which are significantly higher than those of hemangioma (9.3, range: 3.1–41), focal nodular hyperplasia (10, range: 2.9–26) and cirrhotic nodules (11, range: 4.4–49)^104^. In addition to breast cancer and liver cancer, pancreatic tumors are also stiffer than normal pancreatic tissue^105–107^.

However, although tumors are macroscopically stiffer than normal tissues, Plodinec et al.^108^ observed the existence of dispersed softened regions within human breast cancer biopsies and breast cancer tissues in mouse mammary tumor virus-polyoma middle T antigen transgenic mice using an indentation-type atomic force microscopy method, and this finding might be related to metastatic spreading.

### Mechanisms of matrix stiffness in cancer

Matrix stiffness mainly depends on the ECM components and proportion, which is a cardinal phenomenon in many cancers accompanying TME sclerosis. Generally, overabundant collagen and HA are frequently observed throughout the tumor and are responsible for its stiffness^109–111^. However, solid tumors are 3D structures, whose periphery and interior parts show different mechanical characteristics. The interior parts of solid tumors mainly bear compressive stress from tumor cells and a stiffening matrix^112^, while the periphery of the tumor is subjected to tensile stress from the tumor mass and surrounding tissues^112^. Thus, the primary components responsible for stiffness of the periphery and interior of a tumor might be different. For example, excess collagen mostly contributes to the stiffness at the periphery of a tumor^113^. Superfluous HA mainly deposits at the interior of a tumor to counteract the compressive stresses exerted by the periphery layer of the tumor^114^.

Overall, the excessive intratumoral deposition of collagen and HA can be attributed to accelerated synthesis and slowed catabolism. First, some tumor cells can synthesize components of the ECM such as collagen and HA, exhibiting some characteristics of fibroblasts. For example, Fang et al.^115^ showed that type I collagen could be produced by not only fibroblasts but also by cancer cells in the lung and esophageal cancer. Similarly, HA-positive tumor cells can be identified in epithelial ovarian cancer^116^, breast cancer^117^, colorectal cancer^118^, prostate cancer^119^, and gastric cancer^120^. In addition to secreting components of the ECM, cancer cells can also produce enzymes involved in the maturation of ECM proteins. For example, IHC analysis revealed that gastric cancer cells could produce LOX to enhance collagen crosslinking^121^. It is worth mentioning that MMP-2 and MMP-9 are upregulated in human colorectal cancer^122^, and it might partly attribute to the compensatory responses to matrix stiffness so that the local matrix is degraded, which further enhances the motility of cancer cells.

Unfortunately, the production of ECM components and enzymes could be further accelerated when tumor cells receive external signals from growth factors^123^. For example, HAS mRNA transcription can be stimulated by epidermal growth factor (EGF), keratinocyte growth factor (KGF), and PDGF in keratinocytes^124–126^, and these growth factors are frequently overexpressed in cancer.

By contrast, some enzymes catalyzing the degradation of the ECM tend to be suppressed in the TME. For example, hyaluronidase activity decreases in ovarian cancer compared with that in normal ovarian tissue^127^. Moreover, MMP-28, namely epilysin, is significantly downregulated in lung squamous cell carcinoma and adenocarcinoma^128^.

**Fig. 2:**
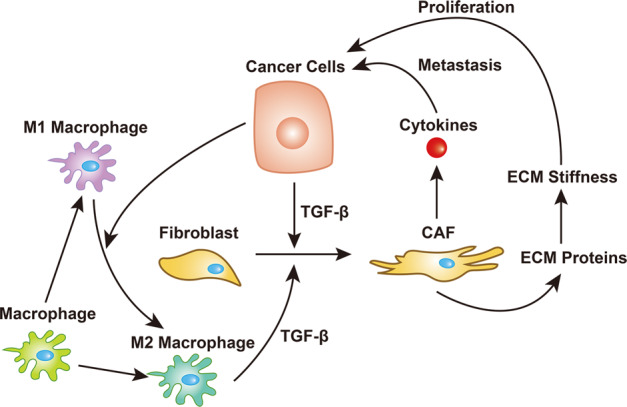
The signaling loop formed by cancer cells, macrophages and fibroblasts contributes to ECM stiffness, in which TGF-β plays a central role

**Fig. 3:**
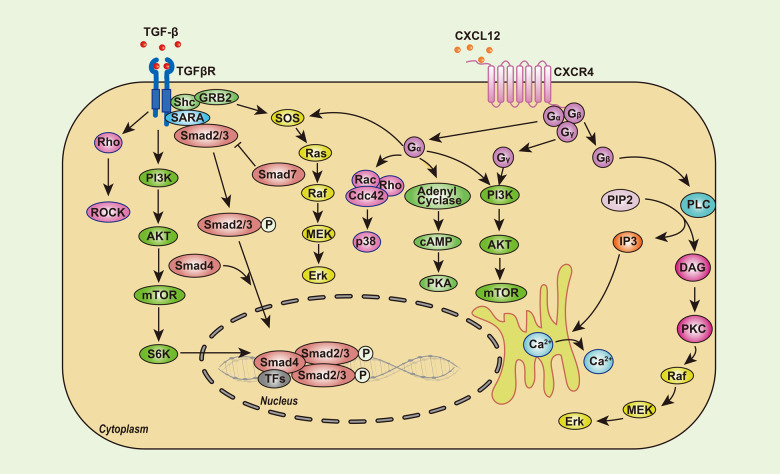
Intracellular signaling network triggered by TGF-β and CXCL12, two critical factors inducing ECM stiffness

In addition to the direct mechanisms described above, tumor cells could secrete growth factors to attract fibroblasts to migrate towards the TME and then transform normal fibroblasts into cancer-associated fibroblasts (CAFs) with a stronger ability to proliferate and promote ECM accumulation. In turn, a stiffened ECM accelerates the growth of tumor cells. Such communication between cancer cells and fibroblasts forms a positive loop feeding the rapid progression of this disease (Fig. 2). During the process of this bidirectional interaction, the TGF-β/Smad2/3 and C–X–C motif chemokine ligand 12 (CXCL12)/C–X–C motif chemokine receptor 4 (CXCR4) signaling pathways are most critical (Fig. 3). Specifically, TGF-β, which could be derived from cancer cells^129^, acts potently on fibroblasts to enhance the synthesis of collagen and fibronectin^130^ as well as chemokines related to tumor promotion, such as CXCR3, CXCR4, C–C motif chemokine receptor 9 (CCR9), CXCL10, CXCL12, C–C motif chemokine ligand 21 (CCL21), and CCL25^131^. These chemokines enhance tumor cell invasion and eventually the occurrence of organ-specific metastases^132^. Moreover, macrophages are lured into the TME by tumor cells^133^ and are further transformed towards the M2 type of macrophages^134^. Next, these M2 macrophages contribute to the activation of CAFs by secreting more TGF-β into the TME^135,136^, feeding more fuel into this positive loop and leading to malignant transformation (Fig. 2).

### The biological effect of matrix stiffness on cancer cells

**Fig. 4:**
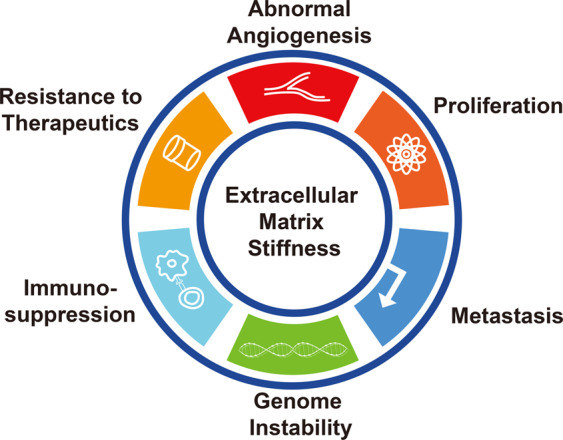
Crucial biological processes affected by ECM stiffness for cancer

A stiffened ECM has fundamental influences on critical biological processes of cancer development, including uncontrolled proliferation, metastasis, angiogenesis, resistance against therapeutics, genome instability (GIN), and immunosuppressive TME (Fig. 4). The mechanisms by which ECM stiffness remodels these key processes will be discussed in-depth in this chapter.

### ECM and growth factors

ECM are important modifiers for the function of many growth factors. Firstly, accumulated ECM can function as a reservoir of growth factors, thus creating a niche with concentrated signaling molecules for the sustained malignant transformation. For instance, Somasundaram et al. proved that PDGF bound to collagen to accumulate in ECM^137^. Heparin-binding growth factor-1, a growth factor associated with angiogenesis, also binds to type I and type IV collagens^138^. What’s more, Paralkar et al.^139^ found that TGF-β bound to type IV collagen in the basement membrane. It is also reported that ECM contains IGFs^140^. Secondly, ECM facilitates the presentation of growth factors to their receptors. For example, glypican-3, a heparan sulfate proteoglycan, promotes the interaction between Wnts and Frizzled through complexing with Wnts, thus stimulating the growth of hepatocellular carcinoma cells both in vitro and in vivo^141^. Interestingly, glypican-3 also directly binds Frizzled through the glycosaminoglycan chains^142^. Furthermore, ECM degradation contributes to the release of growth factors and cytokines^143,144^. During tumorigenesis, MMP-2 and MMP-9 are upregulated in human colorectal cancer^122^, and growth factors released from ECM cleaved by MMPs would promote tumor progression. For example, the VEGF is released when heparan sulfate is degraded, and such process promotes angiogenesis in colorectal carcinoma^145^.

### Effects on the proliferation of cancer cells

**Fig. 5:**
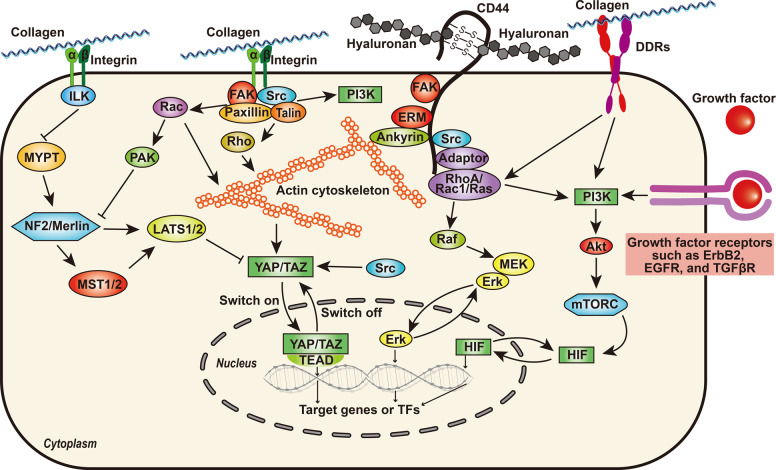
Schematic summary of key membrane receptors recognizing ECM components and their downstream signaling networks

Tumor cells proliferate more slowly in a soft matrix, and matrix stiffness contributes to cancerous proliferation by multiple signaling pathways (Fig. 5)^146–149^. For example, the superfluous collagens bind to integrin on the cell membrane, leading to its allosteric alteration. In addition, the intracellular domain of the integrin β subunit recruits the cytoskeletal protein talin and other cytoskeletal linker proteins, leading to the formation of focal adhesions and activation of Src family kinases (SFKs). Thereafter, assembly of the actin cytoskeleton increases cytoskeletal tension, influencing the myocardin-related transcription factor (MRTF)/serum response factor (SRF) complex. With the help of MRTF/SRF, the signals from the cytoskeleton are transmitted into the nucleus. In addition, activated focal adhesion kinase (FAK) enhances the activity of PI3K^100,150^. Downstream proteins, such as AP-1 (oncogene c-Jun/c-Fos) are activated via Rac/PAK/MEK/ERK, and target of rapamycin is inhibited, eventually contributing to the proliferation of tumor cells. Another important pathway involved in cancer cell proliferation on hard surfaces is the Hippo pathway. This pathway comprises three components, mammalian Ste20-like kinases 1/2 (MST1/2), large tumor suppressor 1/2 (LATS1/2), and yes-associated transcriptional regulator/tafazzin (YAP/TAZ). When matrix stiffness occurs, the activated integrin linked kinase (ILK)-integrin signaling enhances the phosphorylation of myosin phosphatase target subunit 1 and inhibits its activity^151^, leading to the suppression of a signaling cascade comprising Merlin, MST1/2, and LATS1/2^151^. The blockade of upstream signals results in the translocation of YAP/TAZ from the cytoplasm to the nucleus^152^, where they initiate the transcription of genes involved in cell proliferation, such as cyclin D1 and forkhead box M1^153^. In a prospective cohort study in 528 patients with chronic hepatitis B patients, those with a higher liver stiffness (≥10 kPa) showed a significantly higher possibility of developing hepatocellular carcinoma than those showing lower liver stiffness (<10 kPa)^154^.

### Effects on the mobility of cancer cells

Cancer cells also tend to exhibit higher mobility on a stiffened ECM for many reasons. First, the stiffness of ECM can directly activate several signaling transduction pathways involved in cell migration (Fig. 5). For example, Rho/Rho-associated coiled-coil containing protein kinase signaling is activated when cytoskeletal tension is increased in a stiffened matrix^155,156^. Simultaneously, collagen-induced integrin clustering induces the recruitment of focal adhesion signaling molecules, such as FAK, Src, paxillin, Rac, Rho, and Ras, eventually promoting the contraction and progression of cancer cells^157,158^. As mentioned previously, PI3K is also activated when matrix stiffness occurs^150,159,160^, and the downstream molecules AP-1 promotes the migration and invasion of tumor cells. Moreover, the stiffness of the ECM can regulate the expression level of key elements of signaling pathways, indirectly leading to their dysregulation. Gkretsi et al.^161^ reported that Ras suppressor-1 (RSU-1), a cell-ECM protein, is overexpressed in breast cancer cells embedded in stiffer 3D collagen I gels, and silencing RSU-1 led to the inhibition of urokinase plasminogen activator (UPA) and MMP-13, resulting in reduced invasion activity in breast cancer cells.

### Effects on tumor vascularity

Vascularization is an indicator of tumor development and progression^162^. During angiogenesis, vascular loops are formed which link the parental vessels and neovessels, and the matrix of basement membrane is synthesized to reinforce the elasticity and tenacity of blood vessel^163^. Basement membrane, mainly composed of collagen, laminin, fibronectin, often exhibit several abnormal characteristics in tumors, such as disconnection with endothelial cells, disorder of layer and structure, and extensively infiltration into tumor stroma^164^. Vascularization decreases with the increasing matrix density in both collagen and fibrin matrix^165–168^. Matrix stiffness also interrupts endothelial cell–cell junction so that the integrity of barrier breaks down and leads to the leaky vascular^169^. Mechanically, several mechanosensors are in the employ of endothelial cells to convert the mechanical clues into cells so that intracellular biochemical signaling cascades can be activated, such as integrins, actin cytoskeleton, cell-cell adhesion receptors, and other membrane proteins such as ion channels and G-protein-coupled receptors. Taking the mechanical sensor complex of endothelial cells as an example, which is composed of platelet and endothelial cell adhesion molecule 1 (PECAM1), vascular endothelial cadherin (VE–cadherin), and VEGF receptor (VEGFR), PECAM1 functions as a direct mechanosensory, and VE–cadherin serves as an adapter, and VEGFR activates intracellular signaling which consumes energy from GTPase^170–172^. In addition, upregulation of MMP activity in stiffened TME also augments vascular hyperplasia, intrusion, and neovascular branching^169^.

ECM also affects vascularization indirectly through hypoxia. Overgrowth of cancer cells, as well as a structural and functional abnormality of ECM both, contributes to the hypoxia of solid tumor^173^. In turn, the hypoxia circumstance affects the vascularization via multiple mechanisms, the most well understood of which is the activation of hypoxia-inducible factors (HIFs)^174^. Overexpression of HIF-1α has been reported in many malignancies^175,176^. In addition, multiple studies have demonstrated the involvement of HIF-1/VEGF signaling in breast cancer^177^, gastric cancer^178^, bladder cancer^179^, ovarian cancer^180^, and so on.

### Effects on the therapeutic efficacy of chemotherapies, radiotherapies, and targeted therapies

The stiffened matrix in tumors also decreases drug sensitivity, which can partially explain the poor therapeutic efficacy of chemotherapies and immune therapies in many circumstances^181^. First, a stiffened matrix forms physical barriers for drug infiltration into tumor tissue, and researchers have shown that decreasing HA deposited in tumor tissues benefited systemic chemotherapy in colorectal cancer patients with liver metastasis^182^. Second, besides the physical barrier, a stiff matrix compresses micro blood vessels, making it difficult for drugs to access core tumor tissues via the vasculature. Third, ECM stiffness induces hypoxia in the tumor microenvironment^183,184^, further inducing neovascular chaos, resulting in disorganized and perforated intratumoral microvessels. This leaky vasculature negatively influences the transport efficiency of chemotherapeutic drugs^185^. Finally, ECM stiffness is involved in the transformation of tumor cells to cancer stem cells (CSCs), which have the ability to proliferate in a hypoxic environment. Many lines of evidence have shown that CSCs are more resistant to anti-cancer drugs than bulk cells^186–188^.

Matrix stiffness also plays a role in radiation resistance for tumors^189–192^. β1 integrins are upregulated in several types of cancer, such as lung cancer^193^ and colorectal carcinoma^191^, and several studies have demonstrated that the upregulation of β1 integrins contribute to the survival of tumor cells in pancreatic, prostate, glioblastoma, melanoma, and colorectal carcinoma after the treatment of radiation^190,193–195^. Mechanically, the downstream signals of β1 integrins would be activated in response of radiation, such as ILK, FAK, paxillin^194^, c-Jun N2-terminal kinase (JNK), PI3K, and AKT/protein kinase B (PKB)^196^. The activation of PI3K/AKT leads to the radiation resistance^197^, and inhibition of integrin signaling attenuates the insensitivity of cancer cells exposed to radiation^189,196^.

### Effects on genome stability

Recently, some researchers have reported that GIN could also be enhanced by matrix stiffness, whose detailed mechanisms remain under investigation. Some researchers speculate that matrix stiffness would promote cell mitosis, during which spontaneous mutations would accumulate accompanied by fast DNA replication^198,199^. In addition, matrix stiffness increases the probability of nucleus envelope rupture^200^. Nucleus envelope rupture would cause the leakage of nuclear contents into the cytoplasm, such as nucleic acid and nuclease, ultimately causing DNA damage and GIN^201^. Finally, the pore size of the matrix is smaller under stiffer conditions^202^ due to the overabundant deposition of matrix proteins such as collagen^203^. While cancer cells invade, they need to squeeze through smaller pores and undergo more physical damage^204^. Such squeezing movement would isolate some mobilizable nuclear proteins away from DNA^205^, such as DNA repair proteins (e.g., BRCA1), thus increasing the possibility of GIN.

### Effects on infiltrated immune cells and immune therapies

During the development of cancer, immune cells infiltrate into TME and play either anti-tumor and pro-tumor roles. For example, CD8^+^ T cells, CD4^+^ Th1 cells, dendritic cells (DC), natural killer (NK) cells, and M1 macrophages mainly exhibited inhibitory effects on cancer progression, while regulatory T cells (Tregs), CD4^+^ Th2 cells, myeloid-derived suppressor cells (MDSCs) and M2 macrophages mainly show pro-tumor effects^206^. These cells are constantly influenced by the physical, chemical, and biological signals emitted by ECM in TME.

T cells, especially CD8^+^ T cells, are the major players in the immune response against cancerous cells, and they are also the primary targets for checkpoint inhibitor (CPI) therapy currently. The intratumoral ECM contributed to both exclusion and inactivation of T cells in TME by multiple mechanisms. Firstly, the center-axial infiltration of T cells towards chemotaxis gradients is interrupted by the haptotaxis signals from altered ECM^207–209^. Instead of entering TME, some T cells migrate along ECM-rich encapsulation of tumors due to the gradients of substrate rigidity and adhesion molecules such as aligned collagen fibers^210,211^. Such an accumulation of T cells on the periphery of cancerous tissues are reported in many kinds of cancers such as colorectal cancer and lung cancer^212,213^. Secondly, ECM-rich tumors exhibit poor diffusion and hypoxia. The retarded exchange of substances enhances glycolytic metabolism and acidification, which suppresses the activation of T lymphocytes through the specific interaction between V-domain immunoglobulin suppressor of T cell activation and co-inhibitory receptor P-selectin glycoprotein ligand-1 in acidic TME^214^. Moreover, the hypoxic microenvironment increases the production of immunosuppressive factors like TGF-β in favor of Treg differentiation, and it also induces neovascularization with abnormal structure and reduced expression of surface glycoproteins and cell adhesion molecules which are critical for the extravasation of T lymphocytes^215^. Thereby, poor diffusion due to stiffened ECM contributes remarkably to the suppression of anti-tumor responses mediated by T lymphocytes in TME. Thirdly, ECM proteins are directly involved in the regulation of T lymphocytes. For example, CD8^+^ T cells are suppressed by collagen through leukocyte associated Ig-like receptor-1 (LAIR-1)/SH2-containing inositol phosphatase signaling axis in murine cancer models^216^, and high molecular weight HA could enhance the activity of Tregs in vitro^217^. Lung cancer cells also express more PD-L1 in response to ECM stiffness to shut down the anti-tumor immune response mediated by CD8^+^ T cells^218^. Fourthly, stiffened ECM impairs the efficacy of immune therapies. For example, two important obstacles for the application of chimeric antigen receptor (CAR)-engineered T cells therapy in non-hematopoietic malignancies are the low infiltration rate and the immunosuppressive TME, both of which are directly associated with intratumoral ECM accumulation^219^. Similarly, CPI therapies mediated by blocking antibodies are also less penetrable for tumors with high rigidity^220^.

Tumor-associated macrophages are the most frequent immune cells found in the TME^221^. ECM components are able to regulate the polarization of macrophages. For example, collagen and HA are able to drive M2 polarization in vitro^222,223^, while fibronectin enhances cytotoxicity of macrophages against tumor cells, resembling the M1 polarization^224^. Other than macrophages, NK cells have also been shown to be functionally suppressed by transmembrane collagens like Collagen type XVII via LAIR-1^225^.

### Receptors for ECM in normal and cancer cells

Many receptor proteins play an important role in the interaction between ECM and cells, such as integrin, discoidin domain receptors (DDRs), CD44, RHAMM, LAIR-1, and the mannose receptor family, including urokinase plasminogen activator receptor-associated protein^226^. Next, we will focus mainly on integrin, DDRs, CD44, and RHAMM, which are frequently discussed in the context of cancer (Fig. 5).

### Integrin

Integrins are transmembrane heterodimers comprising α subunits and β subunits. In mammals, 18 α subunits and 8 β subunits combine into 24 integrin heterodimers. Among the 24 integrins, four (α1β1, α2β1, α10β1, and α11β1) can bind collagen^226^. Moreover, integrins can bind to various proteins that contain the RGD sequence, such as fibronectin, fibrinogen, laminin, and vitronectin^227–230^. Other than functioning as an anchor, integrins serve as switching points that connect the ECM to the intracellular actin cytoskeleton. Specifically, integrins perceive the ECM mechanical force and then transfer such signals to intracellular proteins such as FAK and Src tyrosine kinases, a process called mechanotransduction. In addition to outside-in signal transduction, integrins also transmit signals from the inside to the outside of the cell when intracellular stimulating molecules bind to β subunits, further influencing the affinity between integrins and the ECM so that cell adhesion, migration, and ECM characteristics might change. For example, Pollan et al.^231^ reported that the adhesion of prostatic cancer cells could be attenuated by silencing CUB domain-containing protein-1 (CDCP1) due to the reduction of inside-out signaling mediated by integrin β1 subunit. Interestingly, the metastatic adhesion of circulating cancer cells may be upregulated by the inside-out signaling via FAK/integrin^232^.

Much research has shown that several integrin proteins are highly expressed in solid tumors and are involved in tumor progression. For example, integrin αvβ3 is upregulated in prostate cancer and promotes cell migration via activation of the PI3K/AKT pathway^233^. Similarly, immunohistochemistry (IHC) analysis conducted by Desgrosellier JS revealed that the positive rate of integrin αvβ3 was significantly higher in metastasis than in primary tumors of pancreatic and breast cancer, and integrin αvβ3 enhanced tumor migration and metastasis by the recruitment of Src kinase^234^. Furthermore, several studies have demonstrated that the upregulation of integrin αvβ3 is correlated with a poor prognosis for patients with oral squamous carcinoma^235–238^, breast cancer^239^, gastric cancer^240,241^, colorectal cancer^242^, pancreatic ductal adenocarcinoma^243^, and cervical squamous carcinoma^244^. Other than integrin αvβ3, integrin αvβ6 is overexpressed in oral squamous carcinoma^235,237^, breast carcinoma^239,245^, gastric cancer^240,246^, pancreatic ductal adenocarcinoma^243,247^, ovarian cancer^248^, colorectal cancer^242,249,250^, cholangiocarcinoma^251,252^, and non-small cell lung cancer^253^.

### Discoidin domain receptors

DDRs can spontaneously bind to collagen and are not regulated by intracellular or extracellular signals. The structure of DDRs includes collagen-binding the discoidin domain at the N-terminus, extracellular juxtamembrane domain, transmembrane domain, intracellular juxtamembrane domain, and tyrosine kinase domain at the C-terminus^254^. There are two types of DDRs, namely, DDR1 and DDR2. DDR1 is commonly expressed in epithelial cells, and DDR2 is generally present in mesenchymal cells such as fibroblasts^255^. Specifically, DDR1 interacts with collagen type I and IV, while DDR2 binds to collagen type I, II, and X. When the collagen-binding discoidin domain interacts with collagen, the conformation of DDRs changes and the phosphorylation of the tyrosine kinase domain leads to the recruitment of adapter proteins (e.g., ShcA and Nck2) to the cytoplasmic domain of DDRs^256^. Both integrin and DDRs can sense ECM stiffness and then transmit this signal into cells. However, ECM cell signal transduction mediated by DDRs is unidirectional, while the one mediated by integrin is bidirectional.

Although heterogeneity remains regarding the expression of DDRs in multiple cancers, many studies have reported that DDRs are overexpressed in cancers. For example, DDR1 overexpression has been observed in breast cancer^257–260^, nonsmall cell lung carcinomas^261–264^, glioblastoma^265^, ovarian tumor^266–269^, endometrial tumors^270^, esophageal carcinoma^271^, head and neck squamous cell carcinomas^260^, hepatocellular carcinoma^272^, cholangiocarcinoma^273^, and prostate cancer^274^. Similarly, DDR2 overexpression is reported in nasopharyngeal carcinoma^275^, cholangiocarcinoma^273^, thyroid cancer^276^, Hodgkin’s lymphoma^277,278^, and acute myelocytic leukemia^279^. In addition, DDR1 overexpression is significantly correlated with a poor prognosis in pancreatic ductal adenocarcinoma^280^, gastric cancer^281^, and nonsmall cell lung cancer^263,282^, while increased DDR2 levels could function as an independent indicator of a worse clinical outcome in breast cancer^283^.

### CD44

CD44 mainly functions as a receptor for HA, collagen, fibronectin, and growth factors. CD44 comprises an extracellular domain, a transmembrane domain, and a cytoplasmic domain^284^, whose isoform heterogeneity is mainly due to the alternative splicing of premRNA and posttranscriptional modifications such as glycosylation (N- and O-glycosylations). HA–CD44 interaction activates multiple cell receptors, such as c-MET, EGF receptor (EGFR), erb-b2 receptor tyrosine kinase 2 (ErbB2), and TGF-β, which then promotes oncogenic pathways. In addition to membrane receptors, the HA–CD44 interaction also activates intracellular signal transducers, such as Grb2, Gab-1, Src, and Rac GTPase families. Thus, many aspects of malignant transformation, such as uncontrolled proliferation, migration and drug resistance could be induced by the HA-CD44 interaction^284,285^. In addition, the binding of lymphocytes to fibronectin is also mediated by CD44^286^, which is pivotal for the infiltration of lymphocytes into the TME. A phase I clinical trial demonstrated that recombinant fibronectin CH296 (FN-CH296) stimulates T cells to achieve strong tumor inhibitory effects in patients with advanced cancer^287^.

Overexpression of CD44 standard (CD44s) and CD44 variant (CD44v) isoforms is widely reported in many types of cancer^288^. In gastric cancer, Yansu Chen et al.^289^ performed a meta-analysis comprising 2403 cases and identified that higher CD44 expression is correlated with a poor overall survival rate and serves as an independent risk factor. A similar observation regarding the prognostic value of CD44 is also reported in other types of cancer, including renal cell carcinoma^290–295^, prostate cancer^296–298^, pancreatic cancer^299–301^, lung cancer^302–307^, breast cancer^308^, colorectal cancer^309–318^, and hepatocellular carcinoma^319–322^. Recently, CD44s and CD44v isoforms have been identified as surface biomarkers for CSCs in pancreatic cancer^323^, salivary gland tumor^324^, laryngeal and nasopharyngeal carcinoma^325–327^, head and neck malignancy^328–335^, gastric cancer^336–341^, colon cancer^312,342–346^, glioma^347–349^, lung cancer^306,350,351^, breast cancer^352^, ovarian cancer^353^, prostate cancer^296,354–356^, and leukemia/lymphoma^357^.

### Receptor for hyaluronan-mediated motility

RHAMM is a unique ECM receptor which lacks a transmembrane domain, and it exhibits both intracellular (cytoplasmic and nuclear) and extracellular (membrane-bound or soluble) localizations^358–361^. RHAMM exhibits highly diverse functions in different subcellular compartments. On cell membrane, HA is the major ligand for membrane-bound RHAMM^362^. RHAMM couples with integral cell surface receptor proteins such as CD44 and growth factor receptors, and HA-RHAMM–CD44 coupling is necessary for the activation of Src/Ras/ERK and FAK/Ras/ERK signaling pathways mediated by CD44^363–366^. Antibodies blocking RHAMM-HA recognition would completely inhibit HA-mediated locomotion, while antibodies blocking CD44-HA recognition failed to change locomotion, suggesting that RHAMM plays a central role for cell motility along HA fibers^367,368^. Moreover, intracellular RHAMM forms direct interaction with MEK/ERK^366^, and it also localizes to multiple subcellular structures including actin filaments, podosomes, the centrosome, microtubules and the mitotic spindle^364,369^. During cell migration, spectrin-α (an actin-associated protein) and RHAMM interact in a complex at the nodes of the actin net to coordinate microtubule polarization^370^. In the centrosome, RHAMM interacts with dynein and maintains spindle pole stability^369^. In the nucleus RHAMM is able to regulate HA-induced activation of the Aurora A kinase (AURKA) by associating with TPX2 (TPX2 microtubule nucleation factor), a critical protein for AURKA recruiting and activating^371^. During mitosis, RHAMM regulates mitotic spindle formation through interacting with tubulin, ERK and TPX2 to recruit and activate AURKA^360,369,372,373^. In mammary epithelium, RHAMM works in concert with TPX2, BRCA1, and AURKA to regulate the apicobasal polarization^374^.

As a dual oncogenic protein promoting proliferation and migration both on cell membrane and intracellularly, RHAMM is overexpressed and correlated with poor prognosis in many kinds of solid tumors, including but not limited to breast cancer^375–377^, colorectal cancer^378,379^, stomach cancer^380^, prostate cancer^381,382^, hepatocellular carcinoma^383,384^, pancreatic ductal adenocarcinoma^385^, lung cancer^386,387^, bladder cancer^388^, oral squamous cell carcinoma^389^, and head and neck cancers^390^. Recently, Choi et al.^391^ reported that RHAMM^B^ isoform was crucial for in vivo metastatic capacity of mouse and human pancreatic cancer while RHAMMA, carrying an extra 15-amino acid-stretch, did not promote metastasis in spontaneous and experimental metastasis mouse models.

## Matrix components as therapeutic targets for cancer

### Therapies targeting collagen

Collagen is one of the most fundamental components in the ECM, the breaking of which could facilitate the penetration of many conventional chemotherapeutic agents and nanoparticles through the barrier of the stiffened matrix in the TME. To alleviate the excessive deposition of collagen in solid tumors with TME sclerosis, several therapeutic strategies have been developed, mostly focusing on the synthesis, degradation, and cross-linking of collagen (Table 1).

**Table 1:** ECM as a therapeutic target in cancer

Drug illustration	Function	Characteristic
Halofuginone	Inhibiting TGF-β signaling pathway and collagen synthesis	Anticoccidial drug
Fresolimumab	Inhibiting collagen synthesis	Monoclonal antibody targeting TGF-β
Collagenases	Collagen degradation	Enzyme
Relaxin	Promoting the synthesis of collagenase	Hormone
MMPs	Collagen degradation	Enzyme
GS-6624	Inhibition of collagen cross-linking	Monoclonal antibody targeting LOXL2
SB-431542 and SB-505124	Inhibiting ALK4/5/7 kinase to block TGF-β pathway	Imidazole analog
Ki26894	Inhibitor of TGFβR1	Small-molecule inhibitor
Candesartan	Angiotensin receptor blockers	Long-acting angiotensin receptor antagonist
4-methylumbelliferone (4-MU)	Inhibitor of HA synthesis	Umbelliferone derivatives
Hyaluronidase	HA degradation	Enzyme
BC-1	Targeting fibronectin for drug delivery	Monoclonal antibody
L19	Targeting fibronectin for delivering drugs and radionuclide	Monoclonal antibody
APT_EDB_	Targeting fibronectin for drug delivery	High-affinity peptides
Vitaxin	Targeting integrin and preventing angiogenesis	Monoclonal antibody targeting integrin αvβ3
Volociximab	Targeting integrin and inhibiting neoangiogenesis	Monoclonal antibody targeting α5β1 integrin
1a-RGD	Targeting the RGD-integrins interaction	Small-molecule integrin antagonist
Cilengitide	Inhibitor of αvβ3 and αvβ5 integrins	Specific peptide antagonist
Imatinib, nilotinib, and dasatinib	Inhibiting tyrosine kinase and DDR signaling pathway	Benzene ammonia pyrimidine derivatives
Bivatuzumab	Blocking CD44-HA interaction	Monoclonal antibody against CD44v6
Verbascoside	Inhibitor for CD44 dimerization	phenylpropanoids
Tranilast	Suppressing TGF-β signaling and expression of extracellular matrix components	Derivative of the amino acid tryptophan
Pirfenidone	Inhibiting TGF-β/Smad and anti-inflammation	Pyridones
Fasudil	Rho-kinase inhibitor	Isoquinoline sulfonamide derivatives
Metformin	Reducing TGF-β signaling, IL-1β, and M2 tumor-associated macrophages infiltration	Biguanides
Dexamethasone	Suppressing angiogenesis and normalizes vessel morphology	Glucocorticoid steroid
Hydroxychloroquine	Macropinocytosis inhibitors	Derivatives of 4-aminoquinoline
All-trans retinoic acid	Retinoic acid receptor agonists	Vitamin
Defactinib	FAK inhibitors and antiangiogenic effect	Benzamides
Ibrutinib	Inhibitor of Bruton tyrosine kinase to interrupt BCR signaling in CLL	Benzene ammonia pyrimidine derivatives
RG7356	Blocking the signaling of CD44 in CLL	Humanized monoclonal antibody for CD44

Considering TGF-β’s crucial role during collagen synthesis, TGF-β signaling is the most promising target to inhibit collagen synthesis^392,393^. For example, an anticoccidial named halofuginone has been shown to reduce collagen synthesis by inhibiting TGF-β signaling in animal models of pancreatic cancer^394^, lung cancer^395^, melanoma^396^, and breast cancer^397^, and tumor migration^396,397^. Moreover, the therapeutic effect of fresolimumab, a monoclonal antibody targeting TGF-β, is currently actively evaluated in several clinical trials (clinicaltrials.gov identifier: NCT01401062 and NCT02581787) to treat cancer^398^. However, a treatment target involving TGF-β should be regarded cautiously because of its extensive roles in both inflammatory and tumorigenesis^399^. In addition to therapies targeting TGF-β, some pilot studies have also found that the classic anti-hypertensive drug losartan contributes to the inhibition of collagen synthesis in both animal models^400,401^ and clinical trials (clinicaltrials.gov identifier: NCT01821729). Although its molecular mechanism still requires further investigation, its safety profile makes losartan a promising choice for designing new therapies targeting collagen synthesis in cancer.

Collagenases can degrade collagen, which could attenuate the stiffness of the matrix and simultaneously contribute to more efficient drug delivery into solid tumors^402^. Due to their chemical nature as proteins, several strategies have been developed to overcome their problem of large molecular sizes to achieve effective transport of collagenases into tumors, such as the oncolytic herpes simplex virus vector^403^ and collagozome (a liposomal formulation of collagenase type I)^404^. For example, Zinger A *et al*. found that the tumor size was reduced by 87% when mice bearing pancreatic tumor xenografts were sequentially treated with collagozome and paclitaxel compared with mice treated with empty liposomes and paclitaxel^404^. More importantly, the researchers found no evidence of the existence of tumor cells in the circulatory system, suggesting that the process of ECM degradation did not trigger tumor metastasis^404^. Interestingly, a hormone named relaxin could improve the penetration of antitumor drugs by indirectly promoting the synthesis of collagenase in osteosarcoma tumor models^405^. In addition to collagenases, a few studies showed that strategies aimed at regulating the quantity or activity of MMPs could also be helpful for cancer treatment, such as marimastat (BB-2516), prinomastat (AG3340), tanomastat (BAY 12-9566), and neovastat^406,407^.

However, two major concerns exist regarding the application of collagenase during cancer therapy. First, the process of collagen degradation might lead to the release of growth factors and cytokines anchored in collagens, which would initiate a cascade of inflammatory signals and tumor progression^408^. Second, the breakdown of collagen might facilitate tumor migration and invasion^409,410^. Therefore, the best time point for the application of such treatment should be considered cautiously and validated experimentally. Theoretically, collagenase-based therapies should be applied to early detected cancer with obvious matrix stiffness that has shown no signs of invasion or metastasis.

Inhibition of collagen cross-linking is another strategy to target ECM stiffness in cancer. For example, studies have found that LOXs are frequently upregulated in many cancers, including thyroid cancer and colorectal cancer^411–413^. Inhibition of LOXs has been shown to enhance chemotherapeutic drug delivery in mouse models of pancreatic cancer^414^ and breast cancer^415^. Theoretically, LOX inhibition might be beneficial during the development phase of cirrhotic cancer. However, it might not work for tumors with an existing mature collagen mesh, greatly limiting its application.

### Therapies targeting TGFβR

TGF-β receptor (TGFβR) is a tetramer which consists of two different transmembrane kinase, namely type I receptor (TGFβR1) and type II receptor (TGFβR2), and both of them have the ability to lead to the phosphorylation of serines, threonines, and tyrosines^416^. When TGF-β binds to TGFβR, the seine at the C-terminal of the adapters such as Smad2 and Smad3 phosphorylates. These adapters, together with Smad4, translocate into the cell nucleus, finally binding to transcription factors so that the transcription of target genes is activated or suppressed^416–418^. TGF-β plays a dual role in cancer. It is a tumor suppressor during the initiating stage early stage of cancer, while it functions as an oncoprotein in advanced stages of cancer^419^. Mechanically, the direct effect when TGF-β binds to TGFβR is proapoptotic^420^, so TGFβR is downregulated or mutant in various types of cancer. However, tumor cells themselves overexpress TGF-β, which is excreted into TME and targets nonparenchymal cells^421^ like fibroblasts and Treg cells. TGF-β promotes fibroblasts to produce ECM components, and enhances the differentiation and function of Treg cells to induce immunosuppressive TME^422^.

Therapies targeting TGF-β have been discussed in the section “Therapies Targeting Collagen” earlier in this review. Small molecules which target TGFβR are widely used in experiments of cancer therapies^423^ (Table 1). SB-431542 and SB-505124 have been shown to suppress proliferation, motility, and vascularization in mice models of glioma and renal carcinoma^424–426^. SB-431542 also enhances the activity of DC and CD8^+^ T cells^423,427^. The fatal weakness of these two inhibitors lies in their instability and low specificity which causes severe systematic toxicity. Ki26894 has been reported to suppress bone metastasis in mice models of breast cancer and gastric cancer^428–430^. Other small molecules that inhibit TGFβR, such as LY-2109761^431–435^, SD-093^436^, SD-208^436^, and LY-580276^436^, have also been tested in various kinds of cancer.

In consideration of the complex role of TGF-β and TGFβR in tumors, the therapeutic strategy targeting them should be really cautious. Agonist of TGFβR directly inhibits the growth of tumor cells, but also promotes stromal cells to produce ECM components and contributes to immunosuppression. As for advanced tumors, inhibition of TGFβR suppresses the metastasis and invasion of tumor. Therefore, a comprehensive understanding of tumor traits, disease stage, and TME are prerequisites when applying a therapeutic strategy targeting TGF-β signaling.

### Therapies targeting AT1R

Angiotensin II type 1 receptor (AT1R) and Angiotensin II type 2 receptor (AT2R) are both receptors for Angiotensin II (AngII)^437^. They belong to the family of G-protein-coupled receptors, which are seven-span transmembrane proteins. AT1R is considered as the leading receptor for AngII to exert vasoconstriction functions, while AT2R tends to be a counter-regulatory factor. The downstream signaling of AT1R, directly or indirectly, includes MAPK, c-Src, Tyk2, Pyk2, Jak2, Ras, AKT, receptor tyrosine kinases, and redox-sensitive transcription factors such as nuclear factor kappa B (NFκB) and HIF-1α^438–441^. The phosphorylation of tyrosine in growth factor receptors, integrins, and adhesion-associated adapter proteins such as paxillin, tensin, and Grb2 all promotes the function of AngII, eventually enhancing the phosphorylation of MEK and ERK1/2 induced by EGFR^442–446^.

AT1R plays important roles in promoting cell proliferation, angiogenesis, and inflammation in TME. Firstly, AT1R activates EGFR in breast cancer^447^ and prostate cancer^448^, contributing to the activation of ERK and signal transducer and activator of transcription 3 (STAT3) phosphorylation, and protein kinase C (PKC) activation, thus promoting the proliferation of cancer cells. Secondly, the activating of EGFR by AT1R leads to the increased expression of VEGF in both cancer cells and endothelial cells, and intratumoral endothelial cells are activated in either paracrine or autocrine manner, which contributes to angiogenesis in TME^449,450^. Lastly, AT1R promotes the transcription of cytokines and chemokines, such as IL-6, IL-12, IL-8, and monocyte chemoattractant protein-1 through activating NFκB and AP-1^451^, thus resulting in inflammation. Dysregulations of AT1R and AT2R has been reported in the breast in situ carcinoma^452^, invasive breast carcinoma^453^, skin squamous cell carcinoma^454^, cervical cancer^455^, ovarian cancer^456^, and prostate cancer^448^.

Angiotensin receptor blockers (ARBs) are widely used as traditional antihypertension drugs, and recent research revealed that they could suppress growth and metastasis of cancer (Table 1). Candesartan, a long-acting angiotensin receptor antagonist, inhibits lung metastasis in mice intravenously injected with 3LL cells^457^. Moreover, tumor growth and angiogenesis are inhibited by candesartan in mouse melanoma model^457,458^ and xenograft models of human prostate and ovarian cancer cells^448,456^. Losartan, another angiotensin receptor blocker, is able to inhibit the release of growth factors like VEGF and suppresses tumor growth of glioma cells both in vivo and in vitro^459^.

### Therapies targeting HA

Similar to collagen, two types of therapeutic strategies targeting HA are under investigation, including the inhibition of HA synthesis and enhancement of HA degradation (Table 1).

4-Methylumbelliferone (4-MU) is an inhibitor of HAS. 4-MU has been shown to suppress the activation of CSCs and attenuate chemoresistance in animal models of ovarian cancer^460^. Moreover, Kohli et al.^461^ demonstrated that liposomes containing 4-MU could potently suppress HA synthesis, eventually facilitating the penetration of more liposome drugs into breast cancer xenografts.

Hyaluronidase has exhibited beneficial effects for diseases such as bladder cancer, brain cancer, and gastrointestinal cancer by degrading HA within the TME^402,462^. A few clinical trials are currently evaluating the therapeutic effects of combining hyaluronidase and chemotherapeutic agents such as gemcitabine and fluorouracil^463,464^. Currently, the long-term effects of hyaluronidase on cancer therapy remain under investigation.

### Therapies targeting fibronectin

The researches regarding the application of fibronectin in cancer treatment are mainly focused on its application as a target for precise drug delivery (Table 1). EDA and extra domain B (EDB) of fibronectin are frequently upregulated in tumor neovasculature^465–467^. Therefore, several targeted cancer therapies have been developed targeting EDB. For example, the murine monoclonal antibody against the cryptic domain adjacent to human fibronectin EDB, BC-1 was fused with murine IL12 (huBC-1-mIL-12) and showed inhibitory effects on various kinds of cancer xenografts in immunocompetent severe combined immune deficiency mice, including colon cancer, skin tumor, and prostate cancer^468,469^. A clinical trial of huBC-1-mIL-12 was conducted, and 46% of patients were in stable condition after 6 or more cycles of treatments^470^. Another antibody that targets EDB, L19, was fused with IL-2 (L19-IL-2) and significantly improved the tumor-inhibitory efficiency of IL-2 in tumor-bearing mice^471,472^. Patients who received L19-IL-2 treatment showed stable condition without treatment-related death during its clinical trial in renal cell carcinoma and melanoma patients^473,474^. Other than cytokines, EDB also serves as the delivery target for antibody-mediated radioisotopes. A fusion protein of L19 and small immunoprotein (SIP) marked with ^131^I not only slowed tumor growth, but also prolonged the survival of mice bearing F9 teratocarcinoma and head and neck carcinoma xenografts^475,476^. In addition, ^131^I-labeled L19-SIP could be applied to visualize the tumor lesions in lymphoma^477^ and prostate cancer^478^ patients. However, no curative effect was observed in these patients. Moreover, EDB binding peptides have also been applied for the delivery of chemotherapeutic agents. Saw et al.^479^ developed APT_EDB_, a novel class of high-affinity peptides targeting EDB, and doxorubicin-containing APT_EDB_ liposomes reduced 55% of tumor size while the free doxorubicin reduced 20% of tumor size in tumor allograft mice model. Similarly, APT_EDB_-decorated nanoparticles encapsulating paclitaxel has been applied for the inhibition of neovasculature in a mice model of glioma tumor, and such modification significantly enhanced the intratumoral accumulation of paclitaxel and prolonged the survival time^480^.

### Therapies targeting sensors of matrix stiffness

Integrin is a promising drug target due to its crucial role in both mechanotransduction and other oncogenic processes for malignancy transformation (Table 1). Integrin α11β1, α5β1, α9β1, and αvβ3 are widely expressed by tumor cells and tumor stromal cells, including fibroblasts, endothelial cells, and tumor-associated macrophages, substantially influencing the characteristics of the TME^481–484^. Many preclinical studies have demonstrated that the inhibition of integrin could strongly suppress disease progression^485^. For example, Vitaxin, a humanized monoclonal antibody targeting integrin αvβ3, showed therapeutic potential in breast, lung, and colon cancer patients by preventing intratumoral angiogenesis during clinical trials^486^. Similarly, volociximab, an antibody that binds specifically to integrin α5β1, also exhibited remarkable therapeutic efficacy in clinical trials involving ovarian cancer, peritoneal cancer, pancreatic cancer and renal cancer patients^487–490^. In addition, Paolillo et al.^491^ found that 1a-RGD, a small-molecule integrin antagonist that targets the RGD–integrin interaction, could augment detachment-mediated anoikis while suppressing cell migration in glioma cancer cell lines. Cilengitide, a specific peptide antagonist targeting the binding between integrin αvβ3 and RGD, shows a good safety profile and clinical improvement for patients with head and neck tumors^492–495^. However, it should be cautioned that the binding of cilengitide with integrin is accompanied by conformation alteration, leading to adverse effects such as agonist-like activities^496^.

Emerging studies have demonstrated the role of DDR1 in cancer progression and metastasis^497–499^ (Table 1). Aguilera et al.^500^ knocked down DDR1 by siRNA and found that migration was inhibited in pancreatic ductal adenocarcinoma cells. The combination of DDR1 inhibitors and classical chemotherapeutic drugs has been reported to reduce the tumor burden in both orthotopic xenografts and autochthonous pancreatic cancer models^500^. Moreover, an in vivo study showed that the knockdown of DDR1 suppressed tumor growth and multiorgan metastasis in breast cancer mouse models^497^. Similarly, in a *KRAS*-mutant lung adenocarcinoma mouse model, inhibition of DDR1 attenuated tumor aggression^501^. The signal transduction triggered by DDRs could be blocked by tyrosine kinase inhibitors (TKIs), such as imatinib, nilotinib, and dasatinib, some of which have been applied as a cancer treatment for more than a decade^502–506^. For example, nilotinib, a second-generation TKI, suppresses tumor metastasis of colorectal cancer cells by inhibiting the DDR signaling pathway in an intrasplenic tumor mouse model^507^. Moreover, lung squamous cell carcinoma patients with a DDR2 mutation were more sensitive to dasatinib than those with wild-type DDR2^508^. Other than TKIs, 3-(2-(pyrazolo[1,5-a]pyrimidin-6-yl) ethynyl)benzamides have been identified as selective DDR1 inhibitors with a relatively low IC50 and could potently attenuate cancer invasion, adhesion, and tumorigenesis in vitro^509^.

Considering the importance of the CD44-HA and RHAMM-HA interactions in tumor cells, they might be promising therapeutic targets for cancer treatment (Table 1). Efforts have been made by many research groups to evaluate the antitumor effect of CD44 antibodies. For example, bivatuzumab (e.g., the first humanized monoclonal antibody against CD44v6 underwent clinical trials), displayed a moderate antitumor effect in patients with advanced squamous cell carcinoma of the head and neck or esophagus^510^. Subsequently, more CD44 antibodies entered clinical trials, such as RO5429083 (clinicaltrials.gov identifier: NCT01358903 and NCT01641250). Moreover, another siRNA-based strategy has been developed to inhibit the mRNA transcription of CD44 or CD44v^511–516^. However, due to the lack of a comprehensive understanding of all CD44 isoforms and the consequences of knocking down a mixture of CD44 isoforms, some challenges persist for the clinical applications of a siRNA-based strategy targeting CD44. Recently, verbascoside has been identified as small molecular-weight inhibitors for CD44 dimerization, and it showed inhibitory effects on the growth of intracranial tumors in a mouse model of glioma^517^. DNA aptamer targeting the HA-binding domain of CD44 also exhibited suppressive effects on the invasiveness of breast cancer cell line MDA-MB-231^518^. Several strategies targeting RHAMM-HA interaction are also under preclinical studies in multiple kinds of cancer. For example, small interfering RNA-mediated suppression of RHAMM has been shown to sensitize lung adenocarcinoma A549 cells to radiotherapy^519^. A soluble peptide containing the HA-binding domain of RHAMM inhibited both proliferation and migration of multiple glioma cell lines^368^. Several shorter blocking peptides (7 to 15mer) for RHAMM-HA interactions have been screened out, but their therapeutic efficacy has not been evaluated in the cancer models yet^520,521^.

### Other therapeutic strategies alleviating matrix stiffness in cancer

A few studies have reported that tranilast^522^, pirfenidone^523^, fasudil^524^, metformin^525^, and dexamethasone^526^ could alleviate matrix stiffness in tumors. In addition, traditional drugs, such as hydroxychloroquine^527^, retinoic acid receptor agonists^527^, and FAK inhibitors^399^, have the potential to attenuate matrix stiffness in tumors. More mechanistic studies are urgently required to exploit these drugs for cancer therapy.

### Cell–ECM interaction as therapeutic targets in leukemia—chronic lymphocytic leukemia as an example

In the previous paragraphs of this review, we mainly focused on the ECM of solid tumors. However, cell–ECM interactions also play nonnegligible roles in leukemia which are often referred to as “liquid cancer”. Even though ECM molecules are relatively sparse in blood, intensive but temporary cell-ECM interactions occur within the bone marrow and peripheral lymphatic organs where hematopoietic cells receive signals for proliferation and differentiation. Similar to solid tumors, collagens, proteoglycans, and glycoproteins form ECM in the bone marrow and lymph nodes. However, unlike solid tumors in which most cancer cells are permanently embedded in stiffened ECM, leukemia cells only form temporary connections with ECM in structured niches within bone marrow and lymphoid organs.

Leukemia can be classified as acute or chronic, according to the degree of cell differentiation, and as myelogenous or lymphocytic, according to the predominant type of cell involved. Generally, leukemia can be categorized as acute lymphocytic leukemia, chronic lymphocytic leukemia (CLL), acute myelogenous leukemia (AML), and chronic myelogenous leukemia (CML). In this section, CLL would be the most frequently used example since remarkable breakthroughs have been made in managing this disease by targeting cell-ECM interactions in the past decade.

In the cortex of lymph nodes, a network of fibroblastic reticular cells (FRCs) secretes type III collagen that produces reticular fibers which are highly stretchable, which allows lymph nodes to enlarge rapidly to accommodate fast-dividing lymphocytes in the circumstances of infections or leukemia. On the apical surface of FRC (the side that faces the cavity where lymphocytes reside), integral membrane proteins (such as vascular cell adhesion molecule, VCAM-1) and other macromolecules tethered to FRC membranes (such as HA) provide abundant anchorage points for lymphocytes and antigen-presenting cells^528–530^. CLL cells actively proliferate inside the lymphoid tissues, but they would stop proliferation during their circulation in blood^531^. Therefore, lymphadenopathy is typically observed in CLL patients. Bruton tyrosine kinase (BTK), a key element of B-cell antigen receptor (BCR) signalosome, plays vital roles for CLL homing and retention in lymph nodes by controlling integrin α4β1-mediated adhesion to fibronectin and VCAM-1, as well as chemotaxis signals mediated by CXCL12-, CXCL13-, and CCL19-induced signaling^532^. CLL patient cells expressed higher BTK mRNA compared to normal B cells^533^. BTK inhibitor Ibrutinib (PCI-32765) treatment can achieve rapid (within days) and sustained reduction of lymphadenopathy accompanied by transient lymphocytosis due to early exiting of CLL cells from lymph nodes^534,535^. Ibrutinib has been approved for patients with previously treated mantle cell lymphoma, CLL, and several other B-cell-related diseases, and more BTK inhibitors are in the pipeline^536^.

Other than BCR signaling, cell-HA interactions in the bone marrow and lymph nodes are also critical for hematological malignancies. For example, CD44v6 expression in diffuse large B-cell lymphoma (DLBCL) correlates with advanced disease stage, and coexpression of any of the CD44 isoforms with RHAMM could identify a subgroup of DLBCL patients with a very poor prognosis independent of the International Prognostic Index^537^. RHAMM-HA interaction promotes directional cell locomotion^538–540^. For example, B cells could mobilize along HA molecules through RHAMM-HA interaction upon activation by chemokines such as CCL21 and IL-8, which is critical for proper B-cell positioning within the lymph nodes^541,542^. RHAMM is highly expressed on B-cell CLL (B-CLL) cells and considered as a promising tumor-associated antigen^543–545^. RHAMM-R3 peptide vaccination could trigger an anticancer immune response in CLL patients^546^. On the other hand, the N-linked glycosylated CD44 variant (CD44v6) forms high-avidity interaction with HA under the stimulation of the CD40 ligand, thus producing an adhesive force to stop CLL mobility, thus retaining CLL cells in specialized compartments of lymph node where they would encounter intensive survival and proliferation-inducing signals^542^. Moreover, engagement of CD44 would activate the intercellular PI3K/AKT and MAPK/ERK pathways, and increase the expression level of myeloid cell leukemia sequence 1 protein to suppress spontaneous and drug-induced apoptosis in CLL cells^547^. These biological functions are in consistent with the observations that CD44 is highly expressed and negatively correlated with prognosis in CLL^548,549^. Zhang et al.^550^ reported that a humanized monoclonal antibody specific for CD44 (RG7356) was directly cytotoxic for CLL cells both in vitro and in vivo, but had little effects on normal B cells.

Other than CLL, CD44-HA interaction plays similar tissue homing and pro-survival roles in AML and CML. Several CD44 antibodies have been shown to suppress AML cells, such as RG7356, HI44a, A3D8, and ARH460-16-2^551–554^. In the Phase I clinical study of RG7356 in AML patients, one complete response (CR) with incomplete platelet recovery (CRp) and one partial response (PR) were recorded, suggesting its potential as an effective therapy for AML^551^. Inhibition of HA synthesis with 4-MU enhanced the anti-proliferative effect of imatinib and doxorubicin in CML^555,556^.

The success of Ibrutinib and RG7356 in hematological malignancies suggests that cell–ECM interactions, as well as the signaling network involved in the tissue homing process, are highly workable and druggable targets for combating hematological malignancies.

## Conclusion and outlook

Cancer is a complex systematic disease involving constant interactions between cancer cells, the ECM, and other cell types present in the TME. Therefore, ECM stiffness plays a vital role during the process of cancer progression, making it a promising therapeutic target for cancer management.

Firstly, ECM serves as a promising and direct therapeutic target for cancer treatment. One of the most successful cases is the application of TKIs in non-small cell lung cancer with EGFR mutation^557^ and chronic myeloid leukemia^558^, which is partially mediated by the blockage of the signal transduction stimulated by DDRs. Therefore, it is urgent for us to identify more cancer-specific ECM targets with the potential of inhibiting cell proliferation, migration, and angiogenesis in the future, thus effectively suppress cancer progression without hurting healthy tissues.

Secondly, intratumoral ECM could be the antigens for the design of tumor vaccine and CAR therapy. Lin et al. showed that vaccination targeting EDA domain of fibronectin enhanced macrophages infiltration, inhibited tumor vasculature, decreased pulmonary metastases^559^ in a polyoma middle T oncoprotein mouse breast cancer model^560^. Zhang et al.^561^ applied CAR macrophage therapy which recognized tumor antigen ErbB2 so that CD147 signaling was simulated and MMPs was increased in a breast cancer mice model. Such CAR therapy softened the ECM, enhanced T cells infiltration, and inhibited tumor growth in mice. However, tumor vaccine and CAR therapy targeting ECM components is relatively more difficult for clinical translation, compared to directly targeting ECM components and related signaling pathways with small molecular compounds or antibodies. The main challenge lies in the scarcity of effective and specific ECM targets in cancer. Another problem is how to accurately predict the host responses to tumor vaccine and CAR therapy in real patients. The complex immune responses in human and animal models in vivo have not been not fully elucidated, which also raises new cautions regarding the safety and workability of translating knowledge obtained in animal models to human patients.

Thirdly, researches of tumor ECM contribute to the development of effective tools for tumor diagnosis and imaging. Traditional imaging heavily relies on radionuclide and single fluorescence, which are limited by the adverse reactions and the accessibility of imaging medium into dense solid tumors. Santimaria et al.^562^ labeled L19, a monoclonal antibody targeting EDB fibronectin, with ^123^I for imaging of colorectal cancer, lung cancer, and brain cancer. Novel imaging materials and methods are springing up these days, and they are safer and more convenient for tumor imaging with lower toxicity. For instance, gold nanoparticles (AuNPs), whose diameter ranges from 1 to 100 nm, possess the traits of sensitivity, specificity, and low clearance, and therefore are designed for coupling tumor-specific RNA^563,564^. Researchers applied second-harmonic generation (SHG) and two-photon excited fluorescence for high contrast imaging of tumors in the deep positions of the body based on the SHG signature of ECM components^565^. In addition, radiomics based on machine learning algorithms are more and more capable of identifying early malignancies, whose combination with detection reagents targeting intratumoral ECM might be a powerful method for the early detection of cancer. What’s more, noninvasive liquid biopsy testing ECM components or fragments could be another direction strategy for cancer at its early stage. Moon et al.^566^ performed liquid biopsy of fibronectin on circulating extracellular vesicles for early detection of breast cancer, and the area under the curves reached 0.70.

Fourthly, ECM normalization can serve as a powerful adjuvant for conventional chemotherapy and immunotherapy. After applying the nanoparticles loaded with pheophorbide a (PPa, a photosensitiser that induced immunogenetic cell death) and NLG919 (an inhibitor of indoleamine 2,3-dioxygenase 1 which enhanced the proliferation of cytotoxic T lymphocytes and inhibited regulatory T cells) targeting MMP-2/9 in ECM, the effect of immunotherapy would be greatly enhanced^567^. The advances of nanotechnology provide opportunities for the spatial and temporal regulation of drug release in TME. Several clinical trials of nanoparticles loaded with albumin-bound (NAB)-paclitaxel is ongoing in early breast cancer^568,569^, advanced biliary tract cancer^570,571^, pancreatic cancer^572^, and so on. Nanoparticle loaded with multiple drugs targeting ECM is now a research hotspot that is promising to achieve low toxicity and high efficiency for their precisely controlled combination of different therapeutic agents. However, some challenges also remain for clinical translation up to now. The most obvious problem lies in the complexity of chemical composition during the in vivo metabolism of such nanoparticles loaded with multiple drugs. Moreover, drug delivery efficiency, tumor-targeting efficacy, drug toxicity, and controllable drug release in time and in place are all issues that need to be considered.

Lastly, there are still Gordian Knots for developing cancer treatment targeting ECM in the field of both scientific research and clinical practices. Currently, there is a lack of materials that can accurately simulate the ECM in vitro. Although Matrigel is widely applied in 3D culture and Gelatin Methacryloyl is frequently used in 3D print, the physical and biological properties, as well as the interaction between different components of ECM is very difficult for an in vitro system to compare with. In addition, ECM stiffness is rarely fully simulated in mice models of solid tumors. Mice have the advantages of quick reproduction, stable genetic background, and low cost. However, it is not very accurate to compare ECM characteristics in tumor-bearing mice whether subcutaneously or orthotopically with real human tumors, due to the great differences in scales and histology. For example, a large proportion of liver cancer is accompanied by cirrhosis, but the subcutaneous and orthotopic liver cancer models, which are commonly used in our drug evaluation, cannot reflect the characteristics of cirrhosis. Dong et al.^573^ either mixed cell suspension with collagen or induced liver cirrhosis with CCl_4_ before the inoculation of xenografts, and the growth of the tumor and the signal transduction pattern altered, resembling human liver cancer specimens accompanied with cirrhosis. Furthermore, systematic assessment is still lacking for the comparison regarding the differences of specific ECM components in different cancer, which is actually the fundamental step for developing strategies of specific therapies and sensitive detection. Last but not least, drug libraries are absent which target ECM components, probably due to the lack of accurate in vitro and in vivo models.

Overall, ECM components contribute greatly to the microenvironment of almost every single cell in the human body, and its dysregulation is closely related to the development and progression of many diseases such as cancer. Over the past two decades, there have been many achievements for the successful application of our knowledge regarding ECM dysregulation in the design of anticancer therapy. With the advances and interdisciplinary integration in cell biology, oncology, material science, and nanotechnology, more and more potent anticancer strategies targeting ECM components and ECM-associated signaling pathways would be translated from basic research to clinical usage, eventually improving the life quality of cancer patients.
